# Case report: Whole-exome sequencing identifies a novel *DES* mutation (p. E434K) in a Chinese family with cardiomyopathy and sudden cardiac death

**DOI:** 10.3389/fcvm.2022.971501

**Published:** 2022-10-04

**Authors:** Yu-Xing Liu, Rong Yu, Yue Sheng, Liang-Liang Fan, Yao Deng

**Affiliations:** ^1^Department of Cardiovascular Surgery, National Clinical Research Center for Geriatric Disorders, Xiangya Hospital, Central South University, Changsha, China; ^2^Department of Cell Biology, School of Life Sciences, Central South University, Changsha, China; ^3^Hunan Key Laboratory of Animal Models for Human Disease, School of Life Sciences, Central South University, Changsha, China; ^4^Department of Anesthesiology, The Second Xiangya Hospital, Central South University, Changsha, China

**Keywords:** hereditary cardiomyopathy, SCD, *DES*, mutation, whole-exome sequencing

## Abstract

**Background:**

Desmin is an intermediate filament protein that plays a critical role in the stabilization of the sarcomeres and cell contacts in the cardiac intercalated disk. Mutated *DES* gene can cause hereditary cardiomyopathy with heterogeneous phenotypes, while the underlying molecular mechanisms requires further investigation.

**Methods:**

We described a Chinese family present with cardiomyopathy and sudden cardiac death (SCD). Whole-exome sequencing (WES) and bioinformatics strategies were employed to explore the genetic entity of this family.

**Results:**

An unknown heterozygote missense variant (c.1300G > A; p. E434K) of *DES* gene was identified. The mutation cosegregates in this family. The mutation was predicted as pathogenic and was absent in our 200 healthy controls.

**Conclusion:**

We identified a novel *DES* mutation (p. E434K) in a Chinese family with cardiomyopathy and SCD. Our study not only provided a new case for the study of the relationship between *DES* mutations and hereditary cardiomyopathy but also broadened the spectrum of *DES* mutations.

## Introduction

Hereditary cardiomyopathy is defined as a primary cardiac disease caused by genetic deficiencies of cardiac muscle (1). Highly heritable but genetically diversely, hereditary cardiomyopathies represent multiple clinical phenotypes, including dilated cardiomyopathy (DCM), hypertrophic cardiomyopathy (HCM), restrictive cardiomyopathy (RCM), arrhythmogenic cardiomyopathy (ACM), non-compaction cardiomyopathy (NCM), and other or mixed phenotypes (2). To date, mutations in over 50 genes have been identified as the causative factor for different forms of cardiomyopathies (3). Proteins encoded by these pathogenic genes, such as sarcomere proteins, nuclear envelope proteins, cytoskeletal proteins, desmosomal proteins, and calcium/sodium-handling proteins of sodium channel, voltage-gated, are strongly linked with the occurrence of cardiomyopathy (4). However, there is no clear genotype–phenotype correlations of cardiomyopathy yet, which poses challenges to clinical diagnosis.

Desmin is an important intermediate filament (IF) protein principally expressed in cardiac, skeletal, and smooth muscle tissue (5). Encoded by the gene *DES*, desmin plays a key role in the mechanical stabilization of the striated muscle sarcomeres and cell contacts within the cardiac intercalated disk (ID) (5). Mutations in *DES* gene have been reported to be associated with skeletal myopathy and different hereditary cardiomyopathies (6). Nevertheless, the exact molecular mechanism of how *DES* mutations lead to these diseases, especially in cardiomyopathies, is not fully understood.

In this study, we described a Chinese family with cardiomyopathy and sudden cardiac death (SCD). Employing whole-exome sequencing (WES) technology and bioinformatics strategies, we identified a novel heterozygous mutation in *DES* gene which may be responsible for the genetic entity of this family.

## Case description

### Clinical features

**FIGURE 1:**
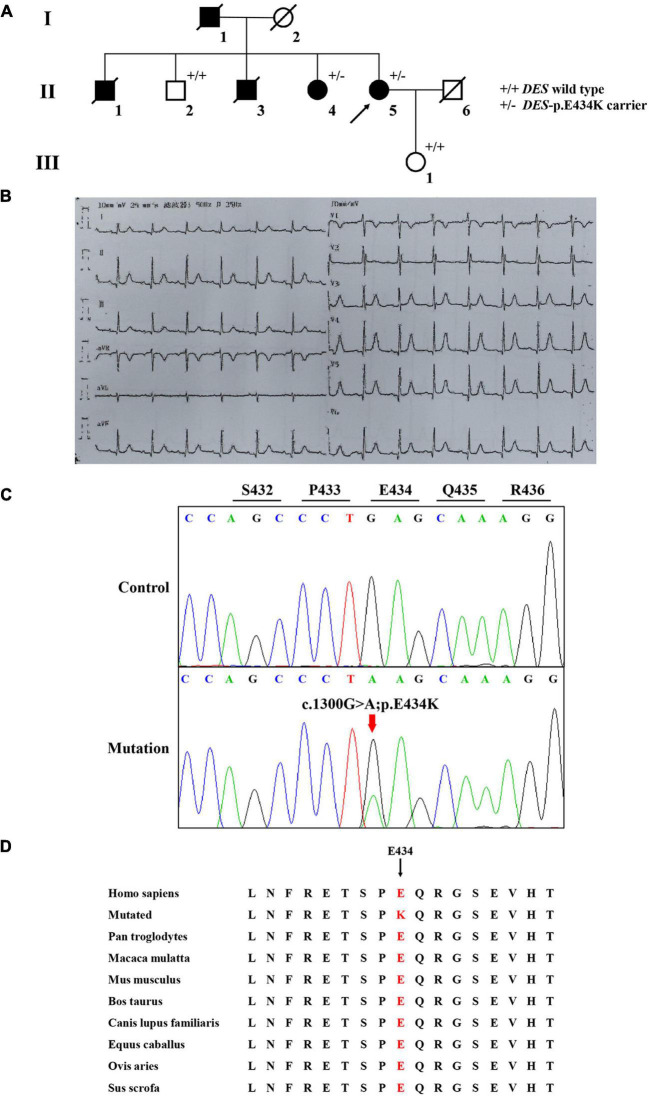
The clinical and sequencing data of this family. **(A)** The genealogy of this family. Squares indicate male family members and circles indicate female members. The black symbols represent the clinically affected members. The white symbols represent unaffected and healthy members. The arrow shows the proband. Crossed-out symbols stand for deceased relatives. ± represents heterozygous *DES*-p. E434K variant. + / + represents a wild type. **(B)** ECG of the proband. **(C)** Sanger DNA sequencing chromatogram detected a heterozygous mutation (c.1300G > A; p. E434K) of *DES* gene in the proband. **(D)** Alignment of multiple DES protein sequences across species. Letters in red show the E434 site is evolutionarily conserved.

A Chinese family with cardiomyopathy and SCD was encountered. The proband (II-5) was a 50-year-old woman from the central south region of China (Hunan Province) (Figure 1A). For more than a decade, she suffered from intermittent chest tightness and palpitation with two syncope episodes experience. According to her medical history, the proband was suspected to be Brugada syndrome in the previous treatment; however, she refused to be further hospitalized for testing and treatment then. During 1 week before her admission, she had increased chest tightness and brief syncope at work, and therefore, she was admitted to our hospital. Physical examination at admission showed high blood pressure (180/130 mmHg). The electrocardiogram (ECG) records showed sinus rhythm, over-load volume of the left ventricle (Figure 1B), but this is not typical for Brugada. The results of ambulatory ECG (AECG) have been shown in Supplementary Figure 1. An echocardiographic assessment revealed the enlargement of left ventricle (LV) and left atrium (LA) (56 and 40 mm, respectively; normal values: <50 and <28 mm, respectively), accompanied by a significant decrease in LV compliance (LVC). Coronary angiography suggested a myocardial bridge and mild stenosis of the left anterior descending artery (Supplementary Figure 2). No myopathies, neurological or bone abnormalities, were found in the proband. Further family history investigation found hypertension in her father (I-1), who died of possible coronary heart disease at age of fifty-two. Her eldest brother (II-1) also suffered from repeated attacks of syncope. Both her eldest brother (II-1) and her third brother (II-3) had SCD. Her sister (II-4) had a history of hypertension. The rest of her family members were healthy and no other malformations were observed in this family (Table 1 and Figure 1A). Given the family history of SCD, the proband received an implantable cardioverter-defibrillator (ICD) and returned for scheduled visits. On the other hand, 200 healthy subjects as described in our previous study were enrolled in this study to exclude polymorphisms (7).

**TABLE 1:** Clinical data of patients in this family.

Subjects	I-1	II-1	II-3	II-4	II-5 (proband)
Sex	M	M	M	F	F
Age (years)	52	48	44	52	50
Hypertension	+	−	−	+	+
Syncope	−	+	−	−	+
SCD	+	+	+	−	−
Sinus rhythm	NA	NA	NA	NA	+
Myocardial bridge	NA	NA	NA	NA	+

F, female; M, male; SCD, sudden cardiac death; NA, not available.

### Genetic analysis

**FIGURE 2:**
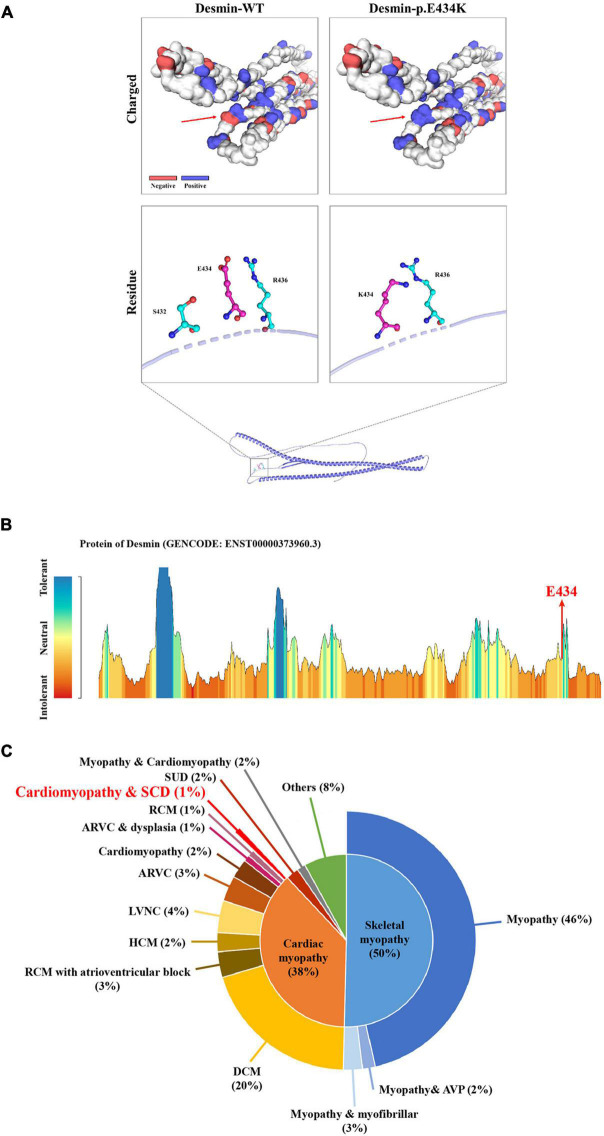
The bioinformatics analysis of mutations. Structure prediction of the mutant protein. The wild-type DES (DES-WT) protein structure and the p. E434K mutant DES (DES-p. E434K) protein structure were predicted by SWISS-MODEL online software. **(A)** Red arrow shows the charged change of desmin protein. In the DES-WT protein, E434 is hydrophobic with S432 and R436. In the DES-p.E434K protein, the hydrophobic effect with S432 disappeared after E434K mutation, while the hydrophobic effect with R436 remained **(B)** MetaDome server was used to identify the intolerant regions in DES. As depicted, the affected nucleotide/residue is located in an intolerant region. **(C)** Overview of all reported phenotypes caused by *DES* mutations. ARVC, arrhythmogenic right ventricular cardiomyopathy; AVP, autophagic vacuolar pathology; DCM, dilated cardiomyopathy; HCM, hypertrophic cardiomyopathy; LVNC, left ventricular non-compaction; RCM, restrictive cardiomyopathy; SCD, sudden cardiac death; SUD, sudden unexplained death.

Whole-exome sequencing yielded 11.7 Gb of data with 99.5% coverage of the target region and 99.1% of the target covered over 10 ×. Data quality control steps, co-segregation, and bioinformatics analysis were performed following the published literature and our previous published studies (8–11). After preliminary screening, variants were further filtered by cardiomyopathy-related genes list as described in our previous study (12). A set of eight variants in eight genes were detected and were further analyzed. Bioinformatics annotations of the eight variants are shown in Table 2. Sanger sequencing was performed on all available family members and showed that a novel heterozygous mutation (c.1300G > A; p. E434K) of the *DES* gene (NM_001927.4) may underlie the genetic factor of this family (Figure 1C). Sanger sequencing confirmed that all living affected members in this family, including the proband (II-5) and her sister (II-4), harbored this heterozygous missense mutation in *DES*. DNA samples of other affected family members (I-1, II-1, and II-3) were unavailable. This mutation was neither identified in the unaffected living family members (II-2 and III-1) (Table 2), nor in our 200 healthy controls. Alignment of desmin amino acid sequences revealed that E434 is conserved in different species (Figure 1D). Modeling of proteins before and after missense mutation was performed by the SWISS-MODEL.^1^ Results revealed that the missense mutation at E434K may lead to the changing of protein surface charge as marked by red arrow in Figure 2A. In the WT desmin protein, E434 is hydrophobic with S432 and R436. In the mutated desmin protein, the hydrophobic effect with S432 disappeared after E434K mutation, while the hydrophobic effect with R436 remained (Figure 2A). Furthermore, MetaDome software^2^ indicated that the affected residue, E434, is located in an intolerant region of desmin (Figure 2B).

**TABLE 2:** Variants identified by WES in this family.

POS	Gene name	Transcript variant	Protein variant	SIFT	Polyphen-2	Mutationtaster	OMIM clinical phenotype	ToppGene function	American college of medical genetics classification	Carrier
Chr11: 46880739-46880739	*LRP4*	NM_002334 c.5513G > A	p. R1838Q	D	D	D	AR, Myasthenic syndrome, congenital	Apolipoprotein binding	PM2; PP3	*II-2*; II-5
Chr2: 220290396-220290396	*DES*	NM_001927 c.1300G > A	p. E434K	T	D	D	AD, Cardiomyopathy or myopathy	Structural constituent of cytoskeleton	PM2; PP1; PP3	II-4; II-5
Chr2: 21255276-21255276	*APOB*	NM_000384 c.1302G > T	p. R434S	D	B	P	AD, Familial hypercholesterolemia	Low-density lipoprotein particle receptor binding	PM2	*II-2*; II-5
Chr2: 71591296-71591296	*ZNF638*	NM_001014972 c.1631G > C	p. R544P	D	D	D	Mu, Autism	RNA splicing	PM2; PP3	*II-2*; II-5
Chr5: 127595212-127595212	*FBN2*	NM_001999 c.8674G > T	p. D2892Y	D	B	D	AD, Contractural arachnodactyly, congenital	Calcium ion binding	PM2; PP3	*II-2*; *III-1*; II-4; II-5
Chr4: 5800368-5800368	*EVC*	NM_153717 c.2153G > A	p. R718Q	T	D	P	AD, Weyers acrofacial dysostosis	Endochondral bone growth	PM2	*III-1*; II-5
Chr17: 78063651-78063651	*CCDC40*	NM_001243342 c.2800C > T	p. R934W	D	D	D	AR, Ciliary dyskinesia, primary	Epithelial cilium movement involved in determination of left/right asymmetry	PM2; PP3	*III-1*; II-4; II-5
Chr10: 60580135-60580135	*BICC1*	NM_001080512 c.2701C > T	p. L901F	D	D	D	AD, Renal dysplasia, cystic	Determination of left/right symmetry	PM2; PP3	*II-2*; II-5

B, begin; D, disease-causing; P, polymorphism; POS, position; T, tolerated; AD, autosomal dominant; AR, autosomal recessive; PP, pathogenic supporting; PM, pathogenic moderate. Italic text represents healthy family members.

## Discussion

In this case report, we reported a Chinese family with cardiomyopathy and SCD. The proband suffered from hypertension and recurrent syncope. Employing WES combined with cardiomyopathy-related gene-filtering, an unknown heterozygous mutation (c.1300G > A; p. E434K) of *DES* gene was detected and might be the pathogenic genetic factor in this family. Of note, the case history investigation of proband’s sister (II-4) revealed that she had hypertension with occasional chest tightness while without a history of syncope. Since recent studies have pointed out that mutations in *DES* gene may cause cardiac myopathies with a broad spectrum of pathological phenotypes within the same family (13). The same is suspected in this family of the present study. Unfortunately, the proband’s sister (II-4) refused to cooperate with further tests because of financial reasons.

Desmin is an IF protein that is prominently expressed in cardiac, skeletal, and smooth muscle tissue (5). As a kind of typical IF proteins, desmin consists of a head domain, a central homologous rod domain, and a tail domain (14). The structure of desmin is related to the assembly mechanism of IF. Different disease-causing *DES* mutations interfere at different stages within this assembly process (15, 16). In our study, the missense mutation (p. E434K) locates in the tail domain that alters the acidic glutamic acid at position 434 to a basic lysine acid. This missense mutation at E434 site may lead to the charged change in desmin protein. The hydrophobic effect of amino acid residues near the mutant site may also be affected (Figure 2A). Although the exact molecular function of the desmin tail domain is not well understood, it is suggested that the tail domain participates in width control of unit length filaments and mediates Ca^2+^- or Mg^2+^-dependent cross-linking (17, 18). Moreover, changes in hydrophobicity of amino acid residues may also affect the formation of a hydrophobic seam, which further leads to filament formation defects (6). Thus, we suggested that the missense mutation (p. E434K) detected in *DES* gene may be disease-causing, in line with the previous studies.

According to published literature, mutations in *DES* gene can cause myopathies or cardiomyopathies with heterogeneous phenotypes (19). Some patients with *DES* mutations may also present a combined skeletal and cardiac myopathy. For cases with cardiomyopathies, the spectrum of cardiac phenotypes associated with *DES* mutations ranges from DCM, HCM, RCM, arrhythmogenic right ventricular cardiomyopathy (ARVC), and left ventricular cardiomyopathy (LVNC). Most of the *DES* mutations were detected in DCM. The associated clinical phenotypes of some mutations overlap significantly. For example, *DES*-p. K144* has been reported in both DCM and HCM. Moreover, *DES*-p. P419S and *DES*-p. R454W have been reported in different cardiomyopathies, including DCM, HCM, ARVC, and RCM (6). These *DES* mutations may be associated with an incomplete penetrance and diverse expressivity. Recent studies have pointed out that mutations in *DES* gene may cause cardiac myopathies with a broad spectrum of pathological phenotypes within the same family (20–24). Some complex or mixed phenotypes had been also reported in rare cases with atypical or unknown cardiomyopathy (6). Nevertheless, it is still not clear why phenotypes caused by *DES* mutations are diverse. A brief review of these reported phenotypes according to the Human Gene Mutation Database (HGMD)^3^ is shown in Figure 2C, which may help the diagnosis of diseases associated with *DES* mutations. It is worth noting that less than 1% of cases reported cardiomyopathy as coexists with SCD, which suggested that the family reported in our study is relatively rare. Of note, the mutation (c.1300G > A; p. E434K) of *DES* gene identified in the present study has not been published, therefore, is considered novel.

## Conclusion

We used WES to explore the genetic entity in a Chinese family with cardiomyopathy and SCD. A novel heterozygous missense mutation (c.1300G > A; p. E434K) of *DES* gene was detected. Our study not only provided a new case for the study of the relationship between *DES* mutations and hereditary cardiomyopathy but also broadened the spectrum of *DES* mutations.

## Data availability statement

The original contributions are included in the Supplementary material and the following database: GSE-Human repository, accession number: HRA002969 (https://ngdc.cncb.ac.cn/gsa-human/browse/HRA002969).

## Ethics statement

The studies involving human participants were reviewed and approved by the Review Board of the Xiangya Hospital of the Central South University in China. The patients/participants provided their written informed consent to participate in this case study. Written informed consent was obtained from the individual(s) for the publication of this care report and any potentially identifiable images or data included in this article.

## Author contributions

RY enrolled the family members. YS performed DNA isolation and sanger sequencing. Y-XL and L-LF performed genetic analysis. Y-XL wrote the manuscript. L-LF and YD supported the project. All authors reviewed the manuscript.

## Footnotes

1
https://swissmodel.expasy.org/

2
https://stuart.radboudumc.nl/metadome/dashboard

3
http://www.hgmd.cf.ac.uk/docs/login.html
