# Supplementary material for: Case report: Whole-exome sequencing identifies a novel DES mutation (p. E434K) in a Chinese family with cardiomyopathy and sudden cardiac death
Source: Front Cardiovasc Med. 2022 Oct 4;9:971501. doi: 10.3389/fcvm.2022.971501 (PMC9580399; doi:10.3389/fcvm.2022.971501)
Supplement: Supplementary file 2 [file Image_1.PDF]

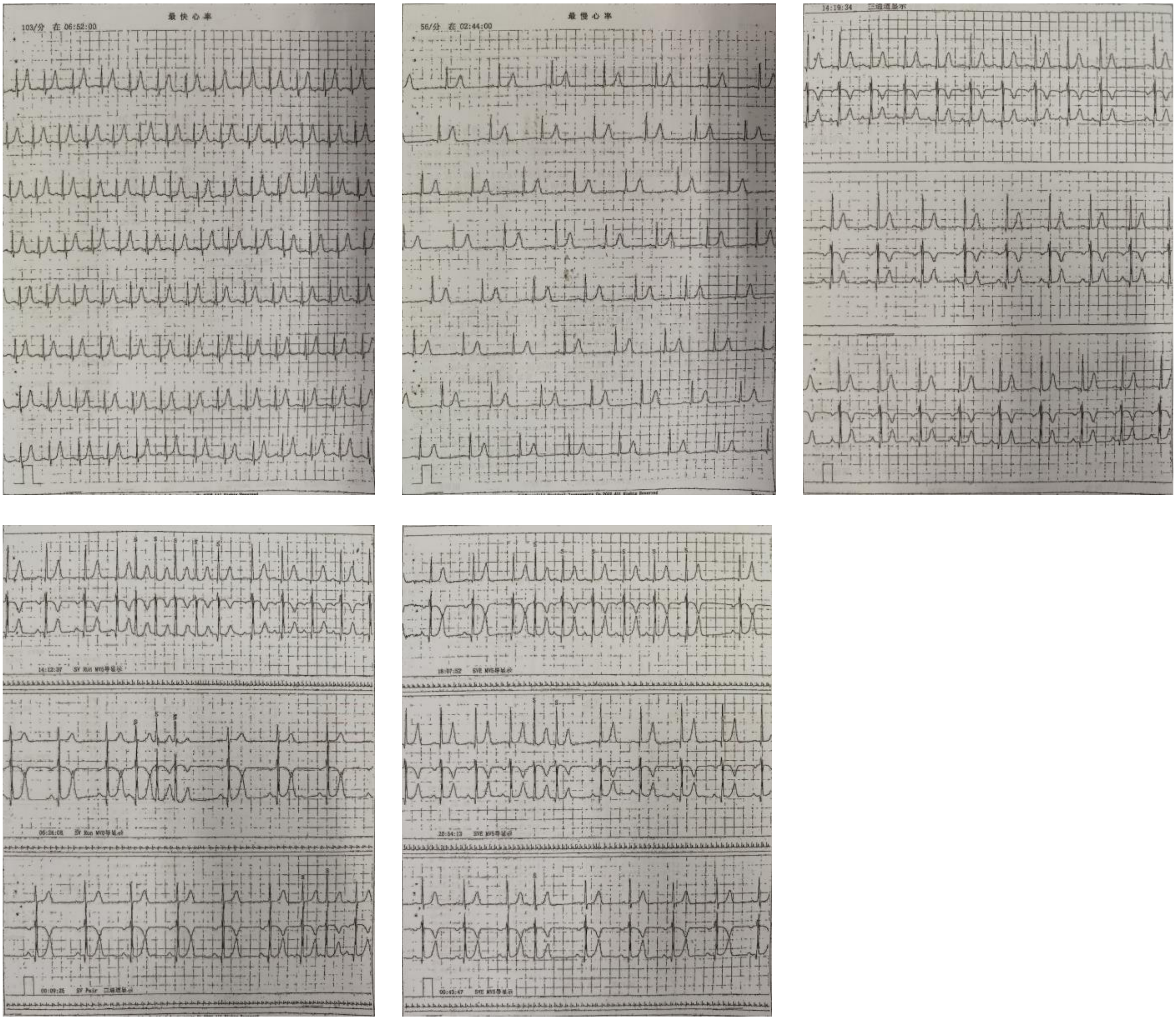

Supplementary Figure 1. Ambulatory ECG (AECG) of the proband.

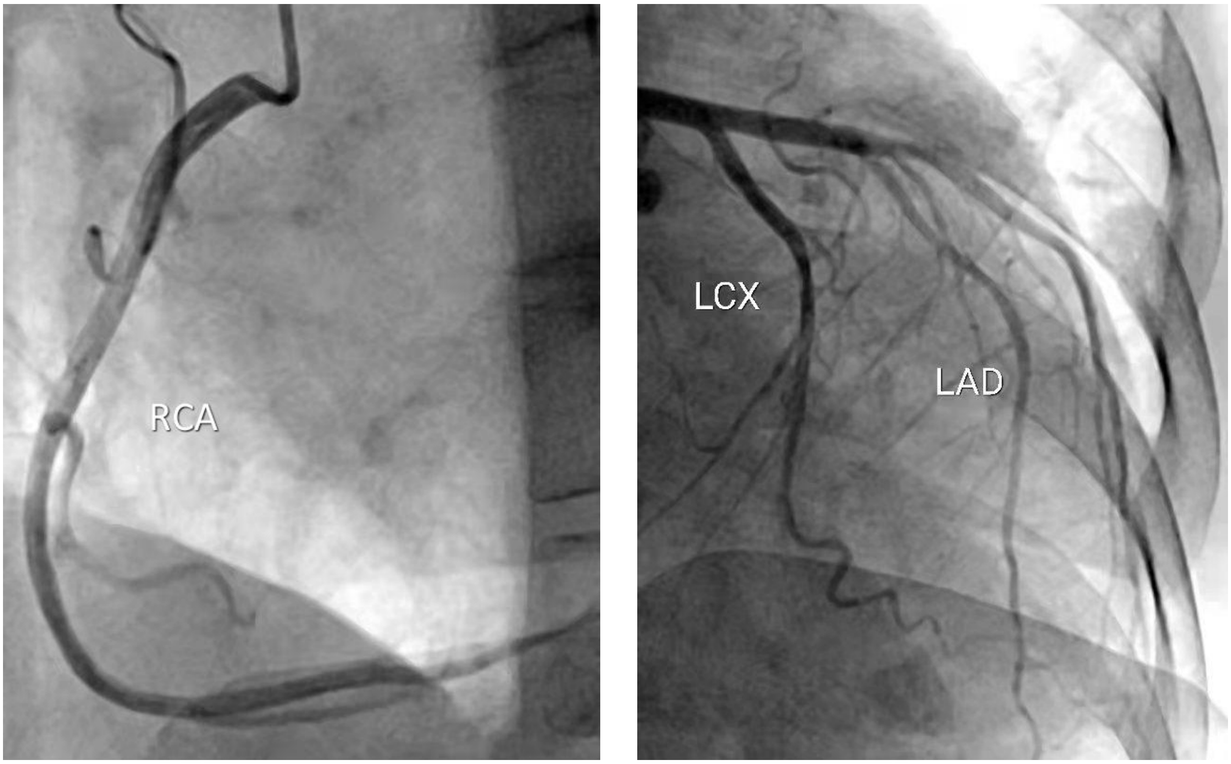

Supplementary Figure 2. Coronary angiogram of the proband.
